# Glutathione transferase theta in apical ciliary tuft regulates mechanical reception and swimming behavior of Sea Urchin Embryos

**DOI:** 10.1002/cm.21127

**Published:** 2013-08-19

**Authors:** Yinhua Jin, Shunsuke Yaguchi, Kogiku Shiba, Lixy Yamada, Junko Yaguchi, Daisuke Shibata, Hitoshi Sawada, Kazuo Inaba

**Affiliations:** 1 Shimoda Marine Research Center, University of TsukubaShimoda, Shizuoka, 415-0025, Japan; 2 Sugashima Marine Biological Laboratory Graduate School of Science, Nagoya UniversityToba, Mie, 517-0004, Japan

**Keywords:** apical tuft, glutathione transferase, ciliogenesis, sea urchin embryo, primary cilia, axonemal dyneins

## Abstract

An apical tuft, which is observed in a wide range of embryos/larvae of marine invertebrates, is composed of a group of cilia that are longer and less motile than the abundant lateral cilia covering the rest of the embryonic surface. Although the apical tuft has been thought to function as a sensory organ, its molecular composition and roles are poorly understood. Here, we identified a glutathione transferase theta (GSTT) as an abundant and specific component of the apical tuft in sea urchin embryos. The expression of GSTT mRNA increases and becomes limited to the animal plate of the mesenchyme blastula, gastrula, and prism larva. Electron microscopy and tandem mass spectrometry demonstrated that the apical tuft contains almost every axonemal component for ciliary motility. Low concentrations of an inhibitor of glutathione transferase bromosulphophthalein (BSP) induce bending of apical tuft, suggesting that GSTT regulates motility of apical tuft cilia. Embryos treated with BSP swim with normal velocity and trajectories but show less efficiency of changing direction when they collide with an object. These results suggest that GSTT in the apical tuft plays an important role in the mechanical reception for the motility regulation of lateral motile cilia in sea urchin embryos.

## Introduction

Cilia and flagella are microtubule-based structures that are well conserved among eukaryotes. The movement of these structures generates fluid flow for the locomotion of single cells or extracellular directional flow over the epithelial surface. Motile cilia and flagella are centered by 9+2 microtubule structures called axonemes. Axonemal dyneins are observed as “arms” attached with nine outer doublet microtubules and are classified by their anchoring positions into outer and inner arm dyneins. The sliding of outer doublet microtubules caused by dyneins is the driving force for oscillatory bending. The central apparatus is composed of radial spokes and central pair microtubules. These structures are thought to be involved in producing planar waveforms by regulating the dynein activity [reviewed in Gibbons, 1981; King, 2000; Porter and Sale, 2000; Mitchell, 2004; Smith and Yang, 2004; Inaba, 2003,2007].

Multicellular organisms possess diverse forms of cilia. Primary cilia are immotile monocilia with mostly 9+0 structures found on most vertebrate cell types [Marshall and Nonaka, 2006]. They function as sensors to perceive chemical or mechanical stimuli and transduce them into intracellular signaling pathways as typified by morphogenesis through the Wnt/planar cell polarity (PCP) pathway [Singla and Reiter, 2006]. Motile 9+0 cilia found on the node of early embryos in mammals are involved in the breaking of left-right symmetry. Dysfunction of these cilia caused by deficiencies in axonemal or intraflagellar transport components leads to diverse diseases, including respiratory defects, male sterility, polycystic kidneys, defects in sensory reception, and randomization of left-right asymmetry [Ibanez-Tallon et al., 2003; Sharma et al., 2008; Sedmak and Wolfrum, 2011].

Diversification of ciliary structures and functions is also observed in invertebrates [Ruiz and Anadón, 1991; Konno et al., 2010]. Before metamorphosis, many of the marine invertebrates undergo a plankton stage in which the larvae swim with motile cilia. In addition to motile cilia on the lateral surfaces, larvae develop longer and less motile cilia, which together are called the apical ciliary tuft. An apical tuft is observed in the embryos/larvae of many marine invertebrates, including sea urchins, bryzoans, polychaetes, mollusks, and cnidarians [Chia and Koss, 1979; Morris and Scholey, 1997; Hadfield, 2000; Yaguchi et al., 2010; Pruliere et al., 2011].

In sea urchins, ciliogenesis starts at the early blastula stage before hatching. At the mesenchyme blastula stage, the embryos show directional swimming by metachronic movement of short lateral cilia. The less motile long cilia at the animal pole, that is, the apical tuft, appear to function as a “rudder” for directional movement [Stephens, 1995]. At later stages, the apical tuft, together with apical ganglions, are considered to function as a sensory organ called the apical organ, and they are suggested to regulate larval swimming and settlement at metamorphosis [Burke, 1978; Nakajima et al., 2004]. However, to the best of our knowledge, no molecular evidence has been obtained to elucidate the functions of the apical tuft in embryonic or larval behavior.

In this study, we examined the differences in protein components between apical ciliary tuft and lateral motile cilia in sea urchin embryos. We first prepared animalized embryos by zinc treatment to obtain sufficient amounts of apical tuft cilia. We found that a glutathione transferase theta (GSTT; previously called glutathione S-transferase theta) is an abundant and specific component of apical tuft. We also found by using an inhibitor of GST that GSTT in the apical tuft appears to play an important role in the mechanical reception for the motility regulation of lateral motile cilia in sea urchin embryos.

## Results

### Glutathione Transferase Theta (GSTT) is a Major Apical Tuft-Specific Protein in Sea Urchin Embryos

**Figure 1:**
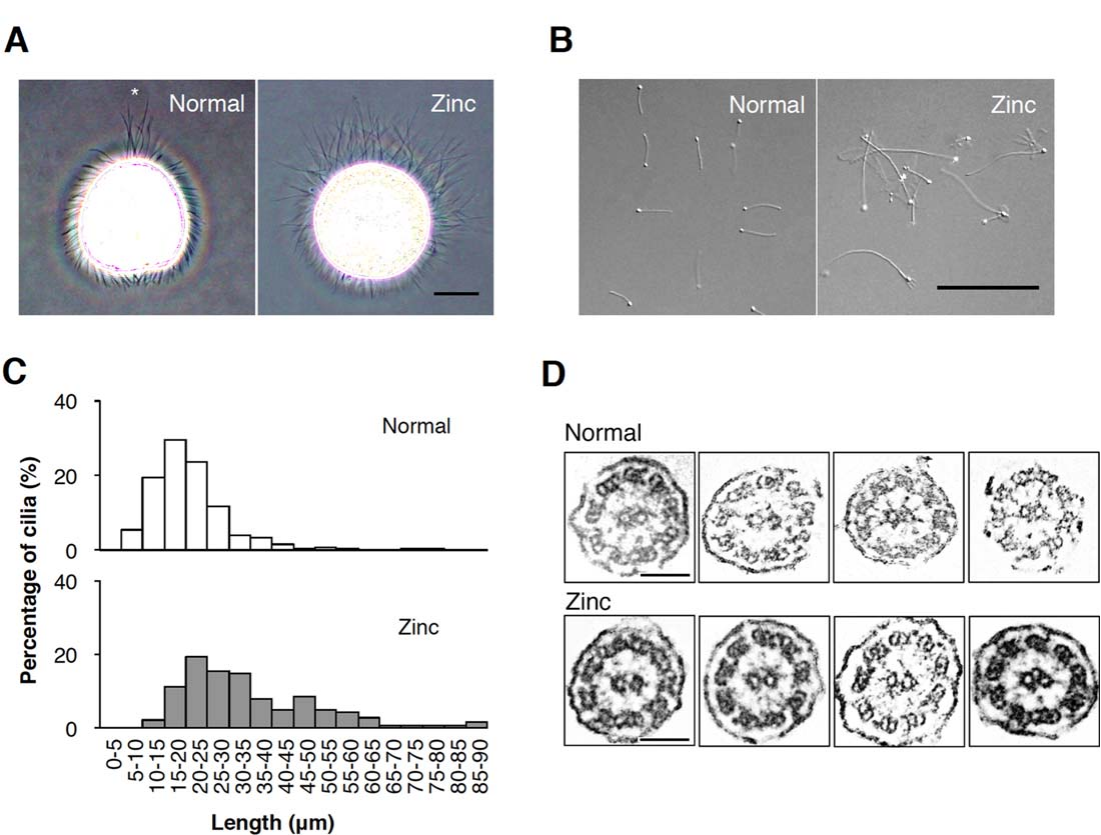
Cilia of normal and Zn-treated sea urchin embryos. (A) Phase contrast microscopic images of normal and Zn-treated embryos at 24- and 36-h post fertilization, respectively. The asterisk indicates the apical tuft. The Zn-treated embryos were animalized and bore long cilia that resemble apical tuft cilia. Bar, 50 μm. (B) Differential interference contrast images of isolated cilia from normal and Zn-treated embryos. Cilia were obtained by deciliation with high-salt seawater. Note that the cilia from Zn-treated embryos are as long as the apical tuft cilia. Bar, 50 μm. (C) Distribution of the length of cilia isolated from normal (open bar) and Zn-treated (closed) embryos. Percentages of cilia in whole isolated cilia are shown. The horizontal bar represents the range of apical tuft cilia directly measured from normal embryos before deciliation. (D) Typical images of cilia from normal (top) and Zn-treated (bottom) embryos by thin-sectioned electron microscopy. Bar, 100 nm. [Color figure can be viewed in the online issue, which is available at http://wileyonlinelibrary.com.]

As the apical tuft is located at the neurogenic animal plate in the sea urchin embryo, the cilia represent only a small number in each embryo. To enrich for apical tuft cilia, we used embryos of a Japanese sea urchin, *Hemicentrotus pulcherrimus*, that were animalized by zinc treatment [Lallier, 1955–1975]. Zn-treatment expands the animal plate region, based on the observation of specific gene expression patterns [Poustka et al., 2007]. The Zn-treated embryos bore a large number of long and less motile cilia, apparently representing those of an apical tuft (Fig. 1A). We treated both normal and Zn-treated embryos with 2X artificial seawater (ASW) to collect cilia [Auclair and Siegel, 1966]. Observation by differential interference microscopy showed a significant difference in the length of isolated cilia between normal and Zn-treated embryos (Fig. 1B). The ciliary lengths of the apical tuft region determined by ankAT-1 expression [Yaguchi et al., 2010] ranged from 35 to 90 μm in the normal embryos (Fig. 1C). From these measurements, the percentages of apical tuft cilia among total cilia in the normal and Zn-treated embryos were estimated as 6.3 and 37%, respectively. Transmission electron microscopic observation showed that major axonemal components, such as outer and inner arms, radial spokes and central apparatus, were present in both cilia from normal and Zn-treated embryos (Fig. 1D).

**Figure 2:**
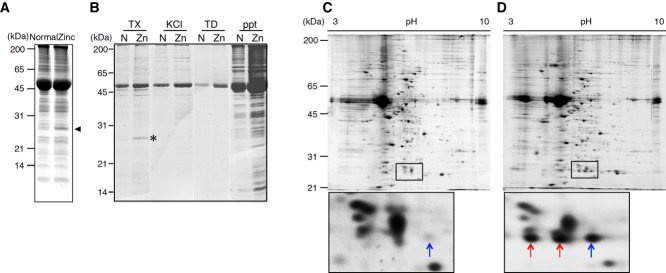
Comparison of ciliary proteins between normal and Zn-treated embryos. (A) SDS-PAGE of ciliary proteins (∼20 μg) from normal (N) and Zn-treated (Zn) embryos. The arrowhead shows ∼25-kDa protein specifically present in cilia from Zn-treated embryos. B, successive extraction of cilia isolated from normal (N) and Zn-treated (Zn) embryos. Cilia were successively demembranated with a buffer containing 0.1% Triton X-100 (TX), a high salt buffer (KCl) and then a low salt buffer (TD). PPt represents the axonemal residues. The ∼25-kDa protein (asterisk) shows present in TX fraction. (C) and (D) 2DE patterns of ciliary proteins from normal and Zn-treated embryos. Horizontal numbers represent pH ranges for isoelectric focusing. The two lower panels show magnified images of the ∼25-kDa regions. The red arrowheads show three spots of ∼25-kDa proteins specifically present in cilia from Zn-treated embryos.

We next compared protein components between cilia from normal and Zn-treated embryos. Sodium dodecyl sulfate-polyacrylamide gel electrophoresis (SDS-PAGE) apparently showed a similar protein pattern between the two samples, except for a 25-kDa protein that was specifically and abundantly contained in Zn-treated embryos (Fig. 2A). During successive extraction of isolated cilia, this protein was extracted with 0.1% Triton X-100 (Fig. 2B), indicating that it was a membrane-bound or cytosolic component. Two-dimensional gel electrophoresis (2DE) also showed a similar protein pattern in major proteins of cilia from normal and Zn-treated embryos, but clear differences were observed mainly in three spots with molecular masses of 25 kDa over broad pI ranges (Figs. 2C and 2D). The most basic 25-kDa spot were also detected in normal embryos in a lesser amount. We also observed differences in other protein spots, including an approximately 100-kDa protein (pI 5.0), a ∼50-kDa protein (pI 5.0), a ∼40-kDa protein (pI 4.5), and a ∼30-kDa protein (pI 4.0).

To identify the 25-kDa proteins showing the most significant differences, we cut out the protein band and spots from SDS- and 2DE-gels, and had them digested by trypsin and subjected to matrix-assisted laser desorption-ionization time-of-flight mass spectrometry (MALDI-TOF/MS). Because the genomic information of the sea urchin *H. pulcherrimus* is not yet available, we used the information from *Strongylocentrotus purpuratus* as the reference database for the mass spectrometry [Sea urchin genome sequencing consortium et al., 2006, SpBases: http://www.spbase.org/SpBase/]. Although the proteins were derived from Japanese sea urchin species, more than 70% of the proteins of randomly selected major 2D spots were identified using the *S. purpuratus* database (data not shown). It turned out that the ∼25-kDa band in SDS-PAGE and all corresponding spots in 2DE showed a significant hit to the gene product SPU_016269. A BLASTP search showed that SPU_016269 encodes a protein similar to *H. sapiens* glutathione transferase theta 1 (or glutathione S-transferase theta 1; GSTT) (E value = 2e-29). We found four gene models for GSTT in the genome of *S. purpuratus*, but they turned out to be identical genes with differently assigned IDs (also see Table1).

**Table 1:** Proteins Specific or More Abundant in Cilia of Zn-Treated Embryos

Gene ID	Molecular weight	pI	Description	Peptide counts in normal embryo (N)	Peptide counts in Zn-treated embryo (Z)	Z/N
SPU_028683	614986.8	8.47	Vitellogenin	–	94	–
SPU_002788	50116.2	4.73	Tubulin beta-2C chain	–	70	–
SPU_016269	19702.9	8.65	Similar to glutathione S-transferase theta 1	–	49	–
SPU_013301	154019	7.04	Vitellogenin	–	43	–
SPU_021662	25318.5	7.03	Similar to glutathione S-transferase theta 1	–	42	–
XP_001197604.1	40442.7	5.62	Hypothetical protein	–	29	–
SPU_027236	30397.8	6.32	Voltage-dependent anion-selective channel protein 2	–	23	–
SPU_010203	38476	7.78	Yolk granule protein; fasciclin-like	–	15	–
SPU_028684	202977.8	6.12	Vitellogenin	–	11	–
SPU_019788	16057.9	10.73	Ribosomal protein L27	–	9	–
SPU_024961	57228.2	8.69	Hypothetical protein-2760 (AnkAT-1)	–	8	–
SPU_010977	31009.9	9.07	Similar to 4930451C15Rik protein	–	8	–
SPU_004813	32740	9.86	ADP/ATP translocase 2	–	7	–
SPU_023066	36279.3	6.13	Chromosome 11 open reading frame 54 protein	–	7	–
SPU_010738	19376.1	5	Vitellogenin	–	7	–
XP_001197263.1	100016.3	7.79	Similar to major yolk protein precursor	–	7	–
SPU_018822	293267.6	5.37	Hypothetical protein LOC757406	–	6	–
SPU_007550	47116.7	8.77	Enta-EF hand domain containing 1-1	–	6	–
SPU_018376	10991.9	7.74	Hypothetical LOC581148, transcript variant 2	–	6	–
XP_001195864.1	32139.5	8.55	Similar to LOC414565 protein	–	6	–
SPU_022072	11369.3	11.2	Histone H4	–	6	–
SPU_007820	15402	11.27	Histone H3.2	–	5	–
XP_001176581.1	19620.9	8.93	Similar to MGC69420 protein	–	5	–
SPU_006767	22189.7	5.96	Sp-Sar1b (Ras superfamily, ARF family)	–	5	–
SPU_024343	13600.8	10.72	Similar to histone H2B-1	–	5	–
NP_999710.2	13615.1	10.43	Histone H2B	–	5	–
SPU_028358	15058.4	6.95	Aldo keto reductase, AKR fragment	–	5	–
SPU_021207	40091.5	6.39	Phosphodiesterase 8A, isoform 1	–	5	–
SPU_010531	51611	4.98	Similar to alpha-tubulin 1C	–	5	–
XP_001177415.1	38534.2	9.16	Yolk granule protein; fasciclin-like	–	5	–
SPU_016270	20652	7.08	Similar to glutathione S-transferase theta 1	1	27	27
SPU_016156	29227.9	9.35	Hypothetical LOC592629	1	18	18
SPU_006495	25100	5.99	Similar to glutathione S-transferase theta 1	5	62	12.4
SPU_019232	10257	6.02	Similar to MGC80929 protein	1	11	11
SPU_018584	36837	9.68	Coiled-coil domain containing 74B-like	1	11	11
SPU_004585	32549	5.64	Hypothetical LOC578897	1	11	11
SPU_000965	25947.4	8.74	Hypothetical LOC585129	1	10	10
XP_001190137.1	15858	5.79	Similar to proteasome-like protein, partial	2	19	9.5
SPU_013919	27343.4	5.49	Fibronectin type III and ankyrin repeat domains 1	2	19	9.5
XP_001176985.1	22241.8	5.31	Hypothetical protein	2	15	7.5
SPU_023486	395128.1	4.3	Fibrocystin L	8	59	7.4
SPU_020730	23740.2	5.19	Similar to thioredoxin family Trp26	2	14	7
SPU_016106	60510	6.3	Several ankyrin repeat protein transcript variant 2	1	7	7
SPU_005791	41687.1	6.1	IQ motif containing K	2	13	6.5
SPU_000253	31918	8.73	Ras-associated protein Rap1-LIKE 1	1	6	6
XP_001181406.1	42417.5	5.3	Imilar to putative ARM-1 protein	1	6	6
SPU_004874	84229.3	9.12	Hypothetical protein-433	1	6	6
SPU_022529	28978.3	5.77	Thioredoxin peroxidase	1	6	6
SPU_000595	50448.4	9.28	Elongation factor 1A	4	22	5.5
SPU_020812	46293.3	4.96	Tubulin, alpha 1C	2	11	5.5
SPU_027647	40643.2	4.89	ATP-binding cassette, sub-family B	1	5	5
SPU_015573	77710.8	5.93	ATP-binding cassette transporter 1	1	5	5
SPU_026062	16406.4	6.84	Similar to RIKEN cDNA 3100002J23 gene	1	5	5
SPU_028434	370512.4	8.17	Similar to dynein heavy chain 14, axonemal	1	5	5
SPU_003378	79189.9	9.03	Zinc finger protein PLAG1	1	5	5
SPU_004774	52312.6	8.64	Hypothetical protein-1543	1	5	5

Proteins with peptide counts from mass spectrometry more than 4 and Z/N more than 5 are listed.

**Figure 3:**
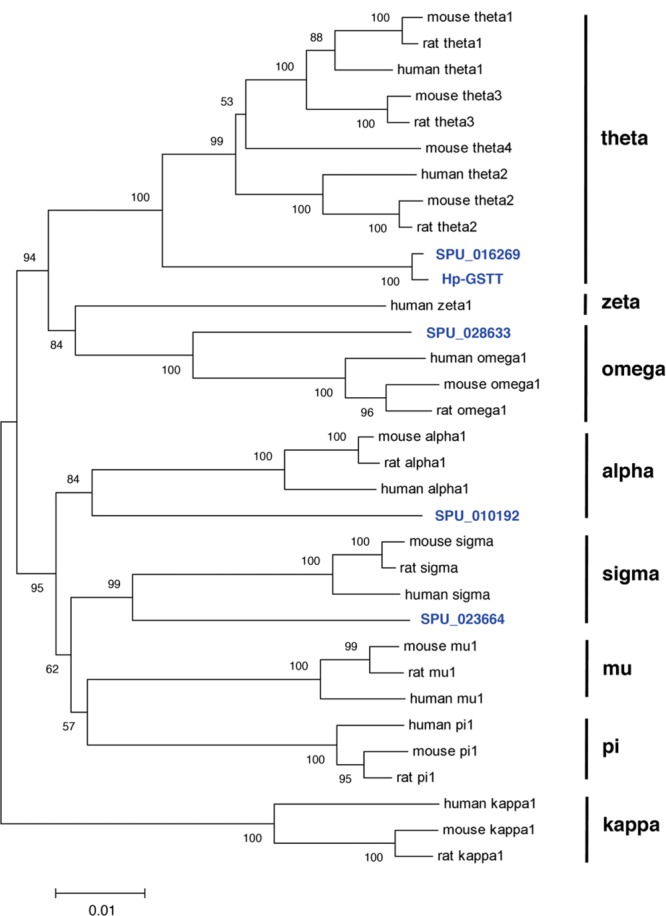
Phylogenetic analysis of GSTs. The consensus phylogenetic tree was constructed by the Neighbor-Joining method from sea urchin and mammalian proteins. The numbers at each node are the percentage bootstrap value of 100 replicates. The accession numbers of the protein sequences used are given in Materials and Methods. Blue letters: proteins of sea urchins. Hp-GSTT was identified in this study with the sea urchin *H. pulcherrimus*. The phylogenetic analysis results indicate that the ∼25-kDa protein identified in the apical tuft is GST theta (GSTT).

Cytoplasmic GST is divided into eight classes in mammals; alpha, kappa, mu, omega, pi, sigma (also known as prostaglandin D synthase), theta, and zeta [Board et al., 1997–2000]. Each class has several isotypes. Our search for GST genes against the *S. purpuratus* database revealed four gene classes with sequence similarities to GST alpha (SPU_010192), omega (SPU_028633), theta (SPU_016269), and sigma (SPU_023664). A molecular phylogenetic analysis showed that the Sp sequences corresponding to the 25-kDa *H. pulcherrimus* proteins abundantly found in Zn-treated embryos are apparently grouped into GST theta (GSTT) (Fig. 3).

### LC-MS/MS Confirmed the Apical Tuft GSTT and Detected More Proteins Specific to Apical Tuft

To determine the molecular differences between apical tuft and lateral motile cilia in more detail, we carried out LC-MS/MS. Several proteins specific to the Zn-treated embryos were identified by a comparison with normal embryos. GSTT was confirmed to be apical tuft-specific, although a small amount was detected in cilia from normal embryos, possibly due to 6.3% cilia in the preparation that appeared to be derived from apical tuft at the animal plate (Table1, Fig. 1). Multiple gene IDs (SPU_016269, SPU_021662, SPU_016270, SPU_006495) showed sequence similarity to GSTT. In addition to GSTT, several redox-related proteins were found to be more abundant in the Zn-treated embryos, containing thioredoxin family Trp26 (SPU_020730), thioredoxin peroxidase (SPU_022529), and thioredoxin reductase 3 (SPU_004780).

Intriguingly the most abundant apical tuft-specific protein was vitellogenin (apolipoprotein B; SPU_028683, peptide count = 94; and other gene IDs included, Table1). In addition, the apical tufts contained several proteins with sequence similarities to histone species (SPU_022072, 007820, 024343, NP_999719, Table1). Other notable proteins found to be specific to the Zn-treated embryos included a factor regulating the length of the apical tuft, AnkAT-1 [Yaguchi et al., 2010] (SPU_024961), a protein defective in a patient with Usher syndrome, Usherin [Weston et al., 2000], a syndecan-binding protein syntenin (SPU_009549) and fibrocystin (SPU_023486). Two tubulin isotypes, beta 2C and alpha 1C, also appeared specific to the apical tufts (Table1, Supporting Information Table SII).

Conversely, we found several proteins specific to normal embryos (Supporting Information Table SI). Aminotransferase class V-1 was exclusively found in cilia from normal embryos, not in those from Zn-treated embryos. Another type of GST isoform, omega, was shown to be specific to cilia from normal embryos. Other interesting proteins found abundantly or specifically in the normal embryos included the actin and microtubule-binding protein coronin (SPU_000974), a 77-kDa echinoderm microtubule-associated protein (SPU_006911), intraflagellar transport protein (IFT144), cytoplasmic dynein2, and a protein similar to serotonin/octopamine receptor family protein 7 (Supporting Information Table SII).

To obtain information on the motile machinery axonemes, we listed the axonemal proteins and their related proteins that were identified by LC-MS/MS in the cilia of normal and Zn-treated embryos in Table2. Major axonemal proteins, such as those of outer and inner dynein arms, radial spokes, central apparatus and other axonemal proteins, were detected in similar quantities between the normal and Zn-treated embryos, suggesting that the apical tuft cilia are potentially motile.

**Table 2:** Axonemal Proteins and the Proteins for Ciliogenesis Found in Cilia from Normal or Zn-Treated Embryos

	Protein	Gene ID	N	Z	Z/N
Outer arm dynein					
	DNAH5 (sea urchin alpha)	SPU_003660, SPU_030226, SPU_000147, SPU_024529, SPU_020621	1122	1250	1.1
	DNAH8 (sea urchin alpha)	SPU_024596, SPU_002110, XP_783106.2, SPU_027840	123	135	1.1
	DNAH9 (sea urchin beta)	SPU_030230, SPU_003404, SPU_017173, SPU_024245	1166	1206	1.0
	DNAH11 (sea urchin beta)	SPU_028599, SPU_028057	15	21	1.4
	IC1 (TNDK-IC)	SPU_007092	99	105	1.1
	IC2 (Chlamy IC69)	SPU_019506	221	186	0.84
	IC3 (Chlamy IC78)	SPU_026533, SPU_005973, SPU_009561, SPU_014776	200	181	0.91
	LC1 (Tctex2)	SPU_013200, SPU_023221	27	34	1.3
	LC2 (LRR)	SPU_018854, XP_001192985.1	27	51	1.9
	LC3 (Tctex1)	SPU_006844, SPU_000795, SPU_007633, SPU_008471	67	93	1.4
	LC4	SPU_008799, SPU_004377	18	35	1.9
	LC5 (roadblock)	SPU_008699	16	24	1.5
	LC6	SPU_024498, SPU_025272	93	137	1.5
	ODA-DC2	SPU_004762	130	111	0.85
	ODA binding protein (Ap58)	SPU_015625	215	165	0.77
Inner Arm Dynein					
	DNAH1	SPU_000013, SPU_030223	492	540	1.1
	DNAH2	SPU_030224	586	718	1.2
	DNAH3	SPU_026539, SPU_012417, SPU_030225, SPU_004622	349	412	1.2
	DNAH6	SPU_030227	316	320	1.0
	DNAH7	SPU_030228, SPU_020747, SPU_010886	582	641	1.1
	DNAH10	SPU_030231	465	467	1.0
	DNAH12	SPU_030232, SPU_003564	382	428	1.1
	DNAH14	SPU_030233, SPU_028434	4	14	3.5
	DNAH15	SPU_030234	366	409	1.1
	IC140	SPU_012809, SPU_013538, SPU_006699	211	215	1.0
	Actin	SPU_009481	247	368	1.5
	p33	SPU_015320	106	109	1.0
	Centrin	SPU_024357, SPU_028660	23	42	1.8
Radial Spoke					
	RSP1	SPU_025942	94	105	1.1
	RSP3	SPU_014801, SPU_012045	179	216	1.2
	RSP4/6	NP_999761.1	19	23	1.2
	RSP9	SPU_005442	161	305	1.9
	RSP10	XP_001182314.1	14	8	0.57
	HSP40	SPU_026705	106	138	1.3
	MORN40/meichroacidin	SPU_010316	21	26	1.2
	CMUB116 (Ciona)	SPU_020748, SPU_026255	38	37	0.97
	Calmodulin	SPU_008000	4	8	2.0
Central Apparatus					
	PF6 (Spag17)	SPU_015915, SPU_000735, SPU_013103	100	98	0.98
	PF16 (Spag6)	SPU_025787	177	277	1.6
	PF20 (Spag16)	SPU_003263	110	124	1.1
	Hydin	SPU_019525, SPU_002460, SPU_013006	131	81	0.62
	CPC1 (central pair complex 1)	SPU_021592	12	12	1.0
	Kinesin, KIF9	SPU_010081	37	32	0.86
Other axonemal proteins					
	Tektin-1	SPU_013841	192	200	1.0
	Tektin-2	SPU_020728	249	225	0.90
	Tektin-3	SPU_023618	213	266	1.2
	Tektin-4	SPU_019591, SPU_008777	239	239	1.0
	RIB43A protein 2	SPU_027579	4	4	1.0
	ODF3 (shippo 1)	SPU_005728, XP_802079.1	50	45	0.90
	PACRG	SPU_004619	264	383	1.5
	PF2 (Dynein Regulatory Complex)	SPU_003865	72	71	0.99
	Coiled-coil domain containing 147 (FAP189/58)	SPU_027352, XP_785052.2	137	103	0.75
ciliogenesis					
	DYNC2H1	SPU_030235	60	50	0.83
	Dynein 2 light intermediate chain	SPU_018582	12	2	0.17
	Dynein light chain 1, cytoplasmic	SPU_018567	29	25	0.86
	Kinesin KIF3B	SPU_022982	27	22	0.81
	Intraflagellar transport 20	SPU_027227	11	6	0.55
	Intraflagellar transport 52	SPU_019590	10	3	0.30
	Intraflagellar transport 74	SPU_018337	4	1	0.25
	Intraflagellar transport 80	SPU_011239	17	6	0.35
	Intraflagellar transport 81	SPU_003223	17	13	0.76
	Intraflagellar transport 88	SPU_020282	8	5	0.63
	Intraflagellar transport 122A	SPU_023605	34	9	0.26
	Intraflagellar transport 139	SPU_002620	39	39	1.0
	Intraflagellar transport 140	SPU_021918, SPU_020159, XP_001192126.1	38	50	1.3
	Intraflagellar transport 144		8	1	0.13
	Intraflagellar transport 172	SPU_013202, SPU_021651, SPU_021760	40	34	0.85
	Bardet-Biedl syndrome 1 protein	SPU_011446	6	5	0.83
	katanin p60 subunit A-like 2	SPU_007977	3	5	1.7

Axonemal components other than tubulins are listed.

### GSTT is Expressed in the Animal Plate of the Normal Embryo

To explore the functions of the apical tuft, we further focused on GSTT, which was abundantly and specifically found in the apical tuft cilia. Because we used Zn-treated embryos to identify apical tuft-specific proteins, it was possible that GSTT was not an intrinsic apical tuft component but was artificially induced by the treatment. To exclude this possibility, we examined the expression pattern of *GSTT* in normal sea urchin embryos. We isolated and sequenced a 1,327-bp cDNA clone for GSTT from *H. pulcherrimus* (termed Hp-GSTT) with an open reading frame encoding 219 amino acids, predicting a molecular mass of 25,256 Da and pI 5.84 (Supporting Information Fig. S1). The molecular mass and pI well matched those that could be estimated by SDS-PAGE and 2DE (Fig. 2).

**Figure 4:**
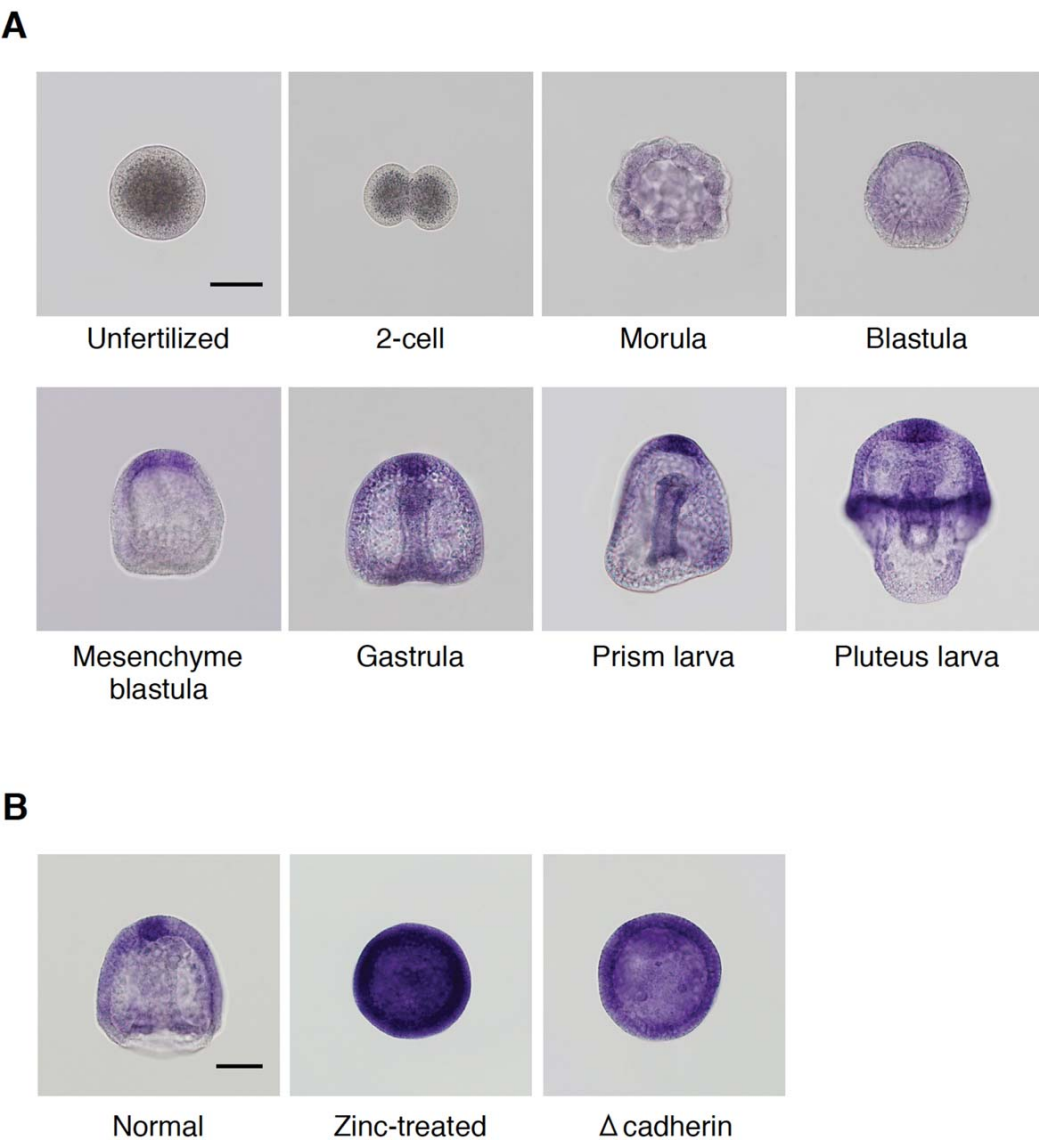
Expression of GSTT gene during the development of sea urchin embryos. (A) Expression patterns by in situ hybridization of several stages of *H. pulcherrimus* embryos. *GSTT* mRNA begins to be highly expressed in the animal plate of mesenchyme blastula and then in the ciliary band of pluteus larva. Bar, 50 μm. B, Expression patterns from in situ hybridization are shown for normal (left), Zn-treated (middle) and Δ cadherin (right) embryos. *GSTT* mRNA was expressed strongly and ubiquitously throughout Zn-treated or cadherin-depleted embryos. Bar, 50 μm.

By using the cDNA as a template, we prepared digoxygenin-labeled RNA probes and performed in situ hybridization. GSTT mRNA was faintly and evenly present until the hatched blastula stage but became increased and limited to the animal plate of the mesenchyme blastula, gastrula, and prism larva. In pluteus larva, the signal became strong at the ciliary band as well (Fig. 4A). Embryos animalized by either Zn-treatment or Δ cadherin injection (Logan et al., 1999) showed strong expression of *GSTT* throughout the entire region of the thickened ectoderm (Fig. 4B).

**Figure 5:**
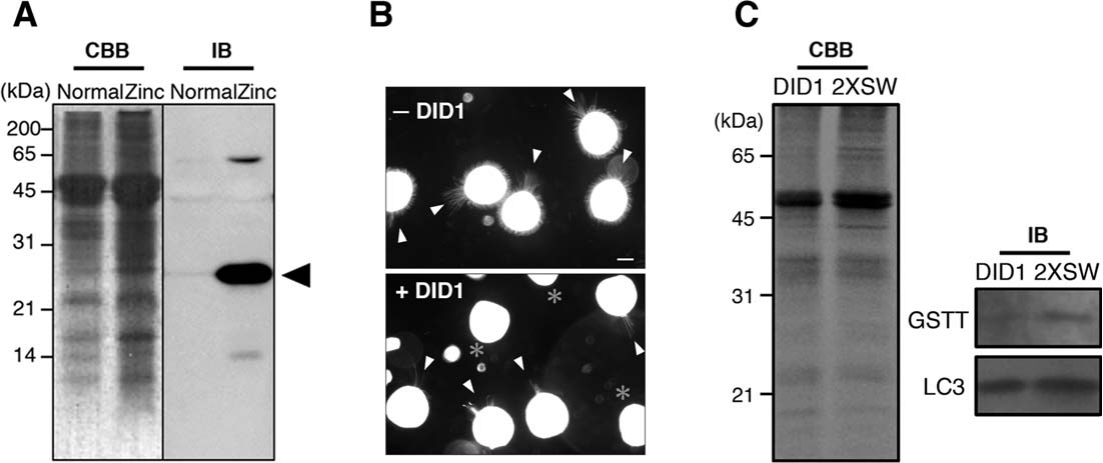
Immunoblots of ciliary proteins with an anti-GSTT antibody. (A) A strong 25-kDa signal corresponding to GSTT (arrowhead) was observed in the Zn-treated embryos, whereas the signal was quite faint in the ciliary proteins from normal embryos. (B) Selective deciliation of lateral motile cilia by DID1. After the treatment of 24-h embryos with DID1, lateral motile cilia and a part of apical tuft were detached. Dark field images of embryos without (−) or with (+) DID1 treatment are shown. Arrowheads show apical tufts. Asterisks represent embryos of which apical tuft is detached. Bar, 50 μm. (C) Lateral motile cilia and a part of apical tuft were isolated by DID1. The rest of apical tuft was isolated by 2X ASW (2XSW) without contamination of lateral motile cilia. GSTT was significantly detected in 2XSW. An antibody against a light chain of outer arm dynein (LC3) was used as an internal control [Hozumi et al., 2006].

The open reading frame of GSTT was subcloned into pET vector in frame, and we prepared a fusion protein and immunized mice to obtain a polyclonal antibody against GSTT. Western blotting against the isolated cilia detected a signal at a 25-kDa protein whose signal was weak in normal embryos but intense in Zn-treated embryos (Fig. 5A). We next tried to isolate apical tuft from normal embryos using dillapiol isoxazoline derivative 1 (DID1) [Semenova et al., 2008]. Although a part of apical tuft was detached, treatment of embryos with DID1 caused selective loss of lateral motile cilia (Fig. 5B). Following treatment of the embryos with 2X ASW resulted in the isolation of the rest of apical tuft without contamination of lateral motile cilia. Western blotting showed that GSTT was concentrated in apical tuft in the normal embryos (Fig. 5C). The result also showed that GSTT was significantly increased in the cilia from Zn-treated embryos.

### Inhibition of GSTT Increases Ciliary Bending in the Apical Tuft

**Figure 6:**
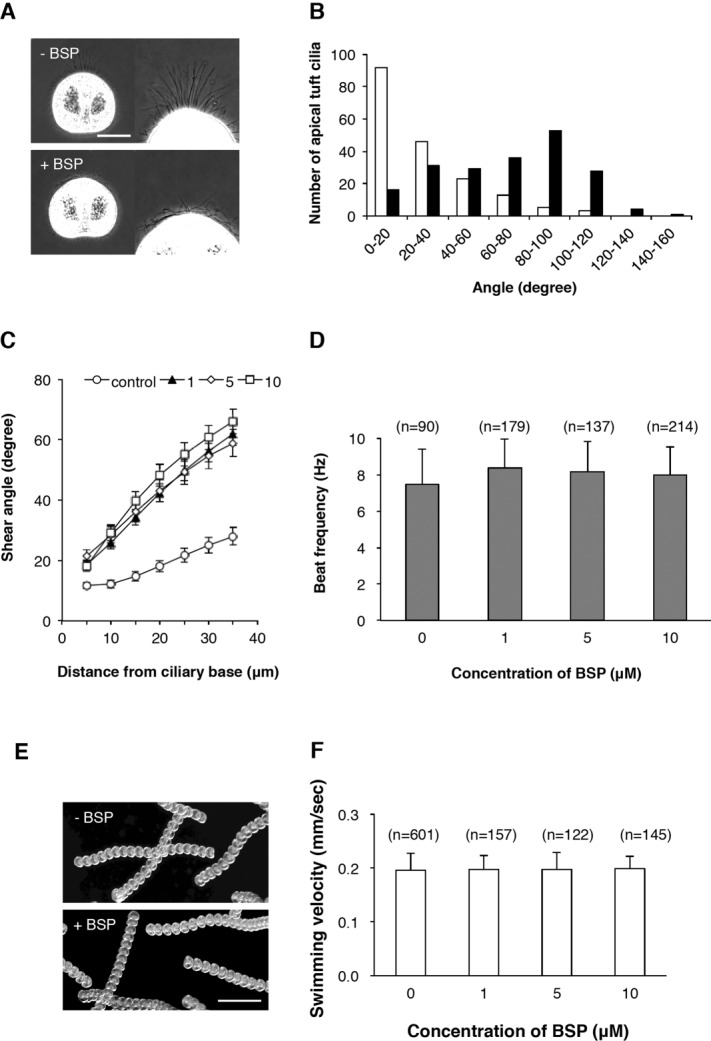
Bending of apical tuft by a GST inhibitor, bromosulphophthalein (BSP). (A) Phase contrast images of embryonic cilia. Top two panels, normal embryo; bottom two panels, embryo treated with 10 μM BSP. Right panels show magnified image of apical tuft regions. Note the bending of apical tuft cilia in the BSP-treated embryos. Bar, 50 μm. (B) Angles of the cilia of apical tuft relative to the animal-vegetal axis in normal embryos (open bar) and in those treated with 10 μM BSP (closed bar). The angle of each cilium was measured from recorded images. (C) Shear angles at various distances from the base of the cilia. Open circles, control (0 μM BSP); closed triangles, 1 μM BSP; open rhombuses, 5 μM BSP; open squares, 10 μM BSP. Bars represents standard error (SE) (*n* = 15). (D) Beat frequency of lateral cilia in embryos treated with several concentrations of BSP. Bars: SE. (E) Swimming speed and trajectories of embryos. Multiple images of normal embryos (top) or those treated with 10 μM BSP (bottom) at 0.05 sec intervals are overdrawn by Bohboh software. Bar: 500 μm. (F) Effect of various concentrations of BSP on the swimming velocity of free-swimming embryos. Bars: SE (*n* = 3).

To investigate the functions of GSTT in the apical tuft, we examined the effects of a potent GST inhibitor, bromosulfophthalein (BSP) [Kolobe et al., 2004], on sea urchin embryos. Treatment of 24- to 28-h embryos (mesenchyme blastula to early gastrula) with BSP induced remarkable bending of the apical tuft (Fig. 6A). The angle of the apical tuft relative to the anterior-posterior axis was greatly increased by the treatment (Fig. 6B). The shear angle showed a more than two-fold increase along the entire length of the cilium (Fig. 6C). The maximum increase in the shear angle was achieved at 1 μM. The BSP treatment did not significantly affect the beat frequency of lateral motile cilia (Fig. 6D).

Ciliary beating is the driving force for larval swimming. To identify the role of GSTT in the control of embryonic swimming behavior, we traced embryonic swimming in a chamber on a glass slide under a stereomicroscope. Both the normal and BSP-treated embryos swam at almost the same speed, with no significant differences in the trajectories (Figs. 6E and 6F).

### Embryos Treated with BSP Showed Normal Negative Geotactic Behavior

**Figure 7:**
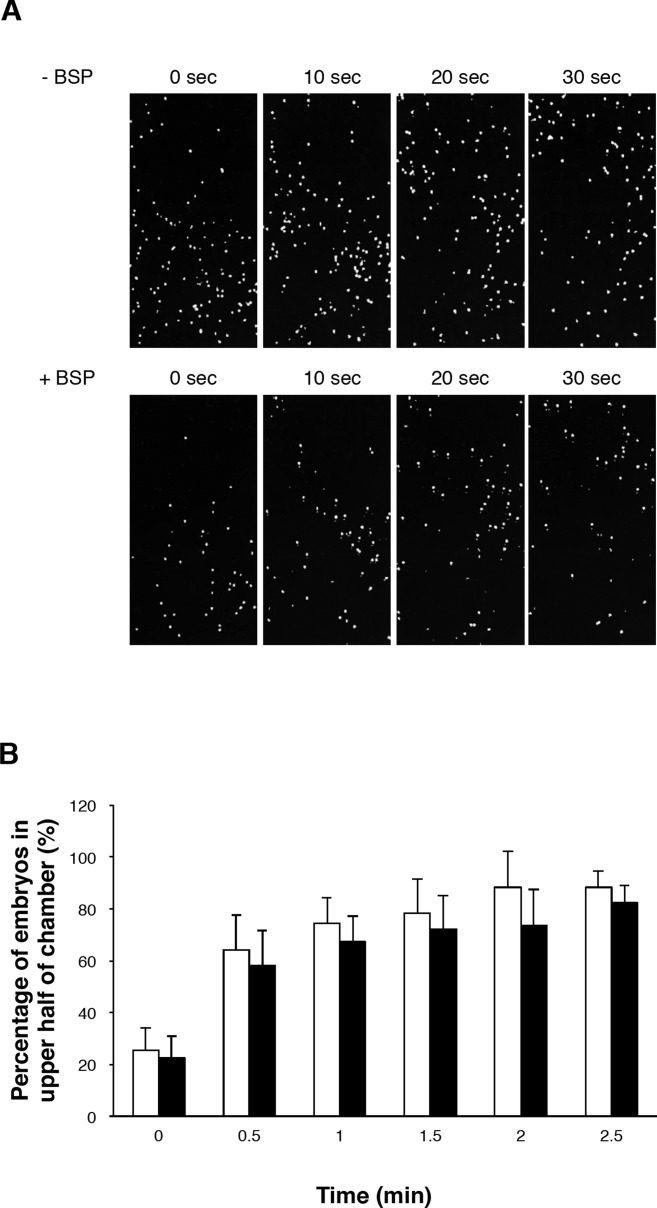
Effect of BSP on the negative geotactic behavior of sea urchin embryos. (A) Sequential images of embryonic movements in a vertically placed chamber. Images from dark-field stereomicroscopy were processed to draw embryos stuck to the wall and background nonembryonic debris. Top, in the absence of BSP; bottom, in the presence of 10 μM BSP. (B) Percentage of embryos that moved into the half top of the chamber against time. Open or closed bar represents embryos in the absence or presence of 10 μM BSP, respectively. Bars: SE (*n* = 6).

Sea urchin embryos/larvae show negative geotactic behavior at the stages from blastula to pluteus [Mogami et al., 1988]. To determine whether GSTT in the apical tuft is involved in the negative geotactic behavior of embryos, we examined the effect of BSP treatment on the embryonic behavior against gravity. A glass slide with a chamber (∼1-mm-thick) was placed vertically, and early blastula embryos were inserted from an entrance at the bottom (Fig. 7A). Normal embryos started to move toward the top of the chamber, and nearly 90% of the embryos reached the upper part of the chamber in 2 min. BSP-treated embryos similarly exhibited negative geotaxis with an only slight difference from the normal embryos (Fig. 7B).

### BSP-Treated Embryos Exhibited Less Escaping Responses against a Mechanical Barrier

**Figure 8:**
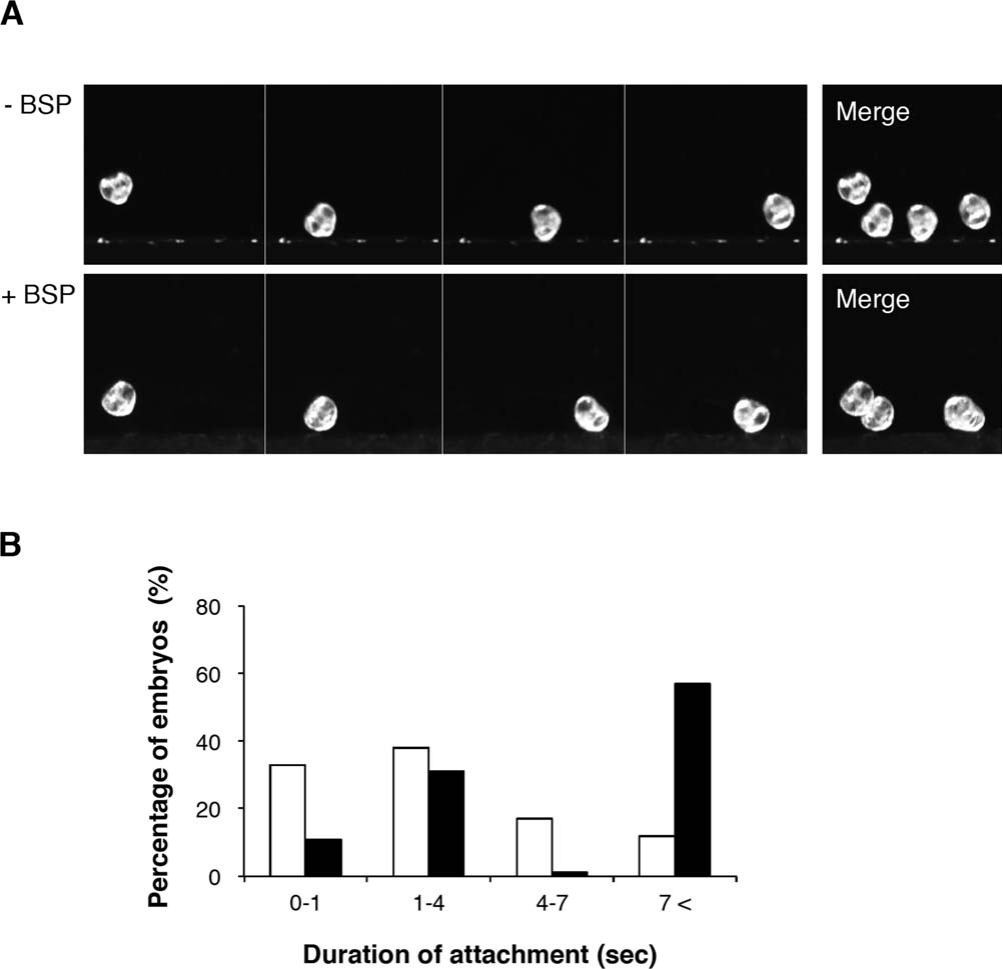
Escaping response of embryos against mechanical barrier. (A) Sequential images of embryos near the wall of the chamber. The bottom dotted lines show the wall of the silicon chamber. The normal embryos changed their swimming direction after colliding with the chamber wall, whereas the BSP-treated embryos were unable to escape and became trapped at the wall. Right panels represent overdrawn images showing trajectories. (B) Distribution of time required for escaping from the wall after collisions, in normal embryos and those treated with 10 μM BSP. Video images from 336 (-BSP) or 356 (+10 μM BSP) embryos were analyzed. The vertical axis represents the percentages of embryos with escaping times in the range of 0–1, 1–4, 4–7, and over 7 sec. BSP-treated embryos with escaping times over 7 sec include those trapped on the wall.

Next we carefully observed the escaping behavior of sea urchin embryos after they collided with the wall of the chamber. The normal embryos efficiently changed their body orientation and swimming direction after collisions with the wall, resulting in an escape from the wall (Fig. 8A, top). More than 70% of the normal embryos escaped from the wall and changed direction to freely swim within 4 sec (Fig. 8B). In contrast, the BSP-treated embryos could not efficiently escape (Fig. 8A, bottom). They stayed on the wall for periods longer than those shown by the normal embryos, without changing their orientation. More than half of the embryos became trapped at the chamber wall (Fig. 8B).

**Figure 9:**
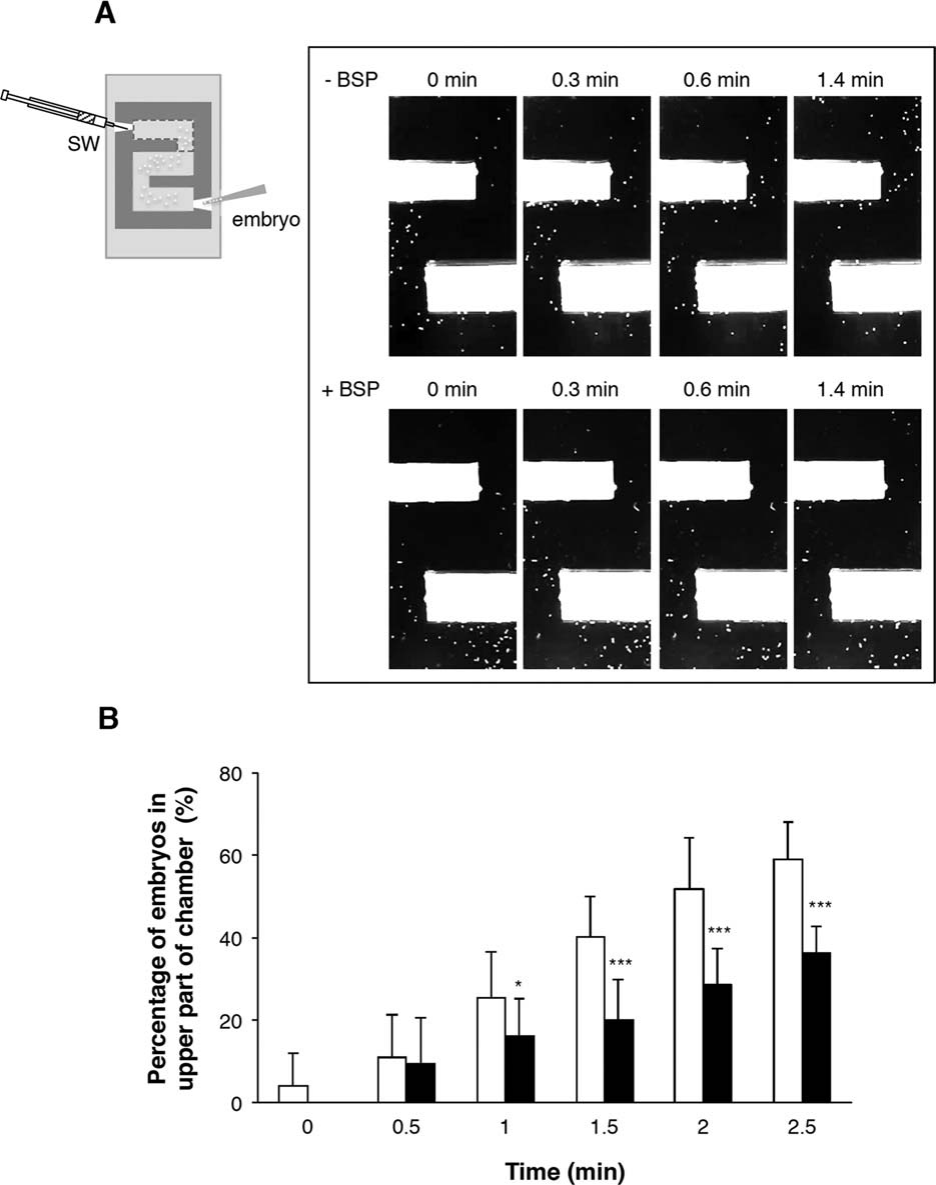
Negative geotaxis in a micro-maze. (A) Left, schematic drawing of the micro-maze. Embryos were introduced to the chamber from the bottom, and excess FSW was removed from the top. Right, sequential images of the distribution of normal (top) and BSP-treated embryos (bottom) in the micro-maze. Images from dark-field stereomicroscopy were processed to draw the background nonembryonic debris. (B) The percentage of embryos that moved into the top compartment of the chamber (shown in red square in A) against time. Open and closed bars represent embryos in the absence and presence of 10 μM BSP, respectively. Bars: SE. *n* = 9 for normal and BSP-treated embryos, respectively. *P < 0.05, ***P < 0.001.

To further explore the role of the apical tuft in the comprehensive response of the embryos to gravity and collision, we made a simple micro-maze to observe the embryos' swimming (Fig. 9A). Embryos were loaded to one side at the bottom of the micro-maze on a glass slide, which was then vertically placed. Nearly 60% of the normal embryos reached the top compartment within 2.5 min (Fig. 9B). Careful observation confirmed that the normal embryos efficiently escaped from the ceilings after collisions so that they could achieve negatively geotactic movement toward the top wall. In contrast, the BSP-treated embryos often became trapped at either of the two ceilings along on the way to the top of the maze, showing significantly lower efficiency in reaching the top compartment compared to the normal embryos (Figs. 9A and 9B).

## Discussion

To obtain an adequate amount of apical tuft to perform a proteomic analysis, we treated sea urchin embryos with zinc. Several studies demonstrated that Zn-treated embryos are animalized and bear long and less motile cilia, similar to the apical tuft [Lallier, 1955–1975; Poustka et al., 2007]. In the present study, the Zn-treated embryos had expanded and thickened ectoderms with characteristics of the animal plate of normal embryos. It was possible that the apical tuft-specific components identified in this study were not present in the apical tuft of normal embryos but were artificially induced by the Zn treatment. The present in situ hybridization and immunoblot analyses clearly showed that at least GSTT was expressed and localized specifically at the animal plate region, suggesting that the cilia isolated from Zn-treated embryos are equivalent to the apical tuft of normal embryos. However, specificity of all the other ciliary proteins identified in Zn-treated embryos is still to be confirmed by other methods.

We found that GSTT was abundantly present in the apical tuft but only present in trace amounts in the lateral motile cilia. Another type of GST, GST omega, was found in the lateral motile cilia, although the amount was not as high as that of GSTT in the apical tuft. Among the multiple types of GST, GSTT is evolutionarily distinct from the other types of GSTs and is conserved among nonmammalian species including plants [Sheehan et al., 2001; Dixon et al., 2009]. GSTT has unique enzymatic properties, including weak binding to a standard GST substrate CDNB (2,4-dinitrochlorobenzene), the bioactivation of dihaloalkanes [Sherratt et al., 1998], and hydroperoxide-reducing glutathione peroxidase activity [Landi, 2000]. In mouse lung, GSTT is localized at the Clara cells and ciliated cells and is suggested to be involved in the detoxification of several carcinogens [Mainwaring et al., 1996].

It should be noted that the sea urchin apical tuft contains abundant amounts of other redox-related enzymes, such as thioredoxin peroxidase (Table1) and thioredoxin reductase (Supporting Information Table SII). Although the exact role of GSTT in the apical tuft was not elucidated in the present study, it is possible that GSTT is involved in the redox-sensitive regulation of apical tuft function.

We observed a significant effect of an inhibitor of GST, BSP, in apical tufts but not significantly in the lateral motile cilia. BSP induced the bending of the apical tufts (Fig. 6), suggesting that GSTT regulates the microtubule-sliding of apical tuft cilia. The bending of the apical tufts seemed to result in less efficiency of escaping behavior when the embryos collided with an object. These results suggest that the apical tuft is involved in the mechanical reception and transduction to regulate lateral motile cilia. We could not exclude the possibility that BSP could affect on other enzymes or other types of GSTs, although BSP did not change the beat frequency of lateral cilia nor the swimming velocity or trajectory of the embryos. Specific knock down of GSTT in the embryos by morpholino antisense oligonucleotides should further clarify the function of GSTT in the control of apical tuft.

Sea urchin embryos show negative geotactic behavior at the stages from blastula to pluteus, which is thought necessary for vertical positioning for feeding [Mogami et al., 1988]. Factors regulating geotactic behavior, which has been widely studied in ciliated protozoa, include uniform density, different drag, and uneven locomotion within a cell [Bean, 1984]. Gravireceptors and rheoreceptors have been posited as possible mechanisms underlying the geotaxis of sea urchin larvae [Gustafson et al., 1972; Strathmann et al., 1972; Mogami et al., 1988], and the apical tuft was one of the candidates. Our present findings indicate that GSTT in the apical tuft is not involved in the negative geotaxis but is involved in the mechanical response to escape from collisions (Figs. 7 and 8). The experiments using a micro-maze clearly showed the role of apical tuft GSTT in the behavior of sea urchin embryos (Fig. 9). It is worth noticing that transient receptor potential (TRP) 11 is localized in flagella and essential for mechanoreception in the avoiding behavior of the unicellular algae *Chlamydomonas* [Fujiu et al., 2011]. It is possible that similar TRP channel is involved in the mechaoreception and avoiding response in multicellular organisms, such as sea urchin embryos. On the other hand, surface scattering of both mammalian sperm and *Chlamydomonas* is primarily governed by direct ciliary contact interaction [Kantsler et al., 2013]. GSTT may participate in the maintenance of mechano-elastic properties of apical tuft. It is still unclear how GSTT is involved in the regulation of apical tuft bending, mechnoreception and control of the motility of lateral cilia. To identify the mechanisms of signal transduction from the apical tuft to lateral motile cilia during an escape after a collision, it is necessary to analyze the detailed changes in the waveforms of apical tuft and lateral cilia before and after collisions.

As the ciliary motility of the apical tuft is much less than that of the lateral motile cilia, we expected the axonemal components for motility to be different between the apical tuft and the lateral motile cilia, but most of the major components of motile axonemes, including those of outer and inner arms, radial spokes and central pair, were identified in both preparations (Fig. 1, Table2). Therefore, it appears that apical tuft cilia are potentially motile and that their reduced motility is the result of regulations inhibitory to the axonemes in apical tuft. We show that GSTT and other redox-related proteins are abundantly present in apical tuft. In *Chlamydomonas* flagella, outer arm dynein is regulated by redox poise [Wakabayashi and King, 2006]. Such a regulation is clearly seen in the changes in flagellar motility when *Chlamydomonas* respond to light stimulation [Wakabayashi et al., 2011]. Furthermore, reactive oxygen species regulates flagellar motility of human sperm [de Lamirande et al., 1997; de Lamirande and Gagnon, 2003]. Taken together, it is possible that the motility of apical tuft is suppressed by redox poise through the action of GSTT. The physiological reason for less motility of apical tuft, however, is still unknown.

We found other proteins specifically present in either lateral motile cilia or apical tuft. Proteins specifically found in apical tuft include GSTT and other redox-related proteins, as well as those suggested to be related to primary cilia and sensory cilia such as fibrocystin [Wang et al., 2007; Harris and Torres, 2009] and usherin [Bhattacharya et al., 2002; Pearsall et al., 2002; Liu et al., 2007]. These features strongly suggest that apical tuft functions as primary cilia or sensory cilia to transmit extracellular signals to the lateral motile cilia. Although the present study suggests that apical tuft GSTT regulated mechanical reception and the regulation of lateral motile cilia, it is not clear how the mechanical signal at apical tuft is transmitted to the lateral motile cilia to regulate the motility and alter the behavior of embryos. Further works to knockdown GSTT and other apical tuft-specific components should shed light on the function of apical tuft in the regulation of ciliary movement and embryonic/larval behavior.

## Materials and Methods

### Chemicals, Reagents, and Solutions

Immobilized pH gradient strips and buffers were purchased from GE Healthcare (Buckinghamshire, UK). Ammonium bicarbonate and Triton X-100 were purchased from Sigma-Aldrich (St. Louis, MO). Trypsin was purchased from Promega (Madison, WI). Molecular weight standards for SDS-PAGE and 2DE were purchased from Bio-Rad (Hercules, CA). DID1 was kindly provided by Dr. Victor Semenov, Zelinsky Institute of Organic Chemistry, Russian Academy of Sciences, Moscow, Russia. All other reagents were purchased from Wako Pure Chemical (Osaka, Japan) or Nacalai Tesque (Kyoto, Japan). The ASW was comprised of 423.0 mM NaCl, 9.0 mM KCl, 9.27 mM CaCl_2_, 22.94 mM MgCl_2_, 25.5 mM MgSO_4_, and 2.14 mM NaHCO_3_ (MBL seawater).

### Handling of Sea Urchin Embryos

Japanese sea urchins, *H. pulcherrimus*, were collected from around the Shimoda Marine Research Center, University of Tsukuba, Research Center for Marine Biology, Tohoku University, and the Marine and Coastal Research Center, Ochanomizu University. The gametes were collected by an intrablastocoelar injection of 0.5 M KCl. After insemination, eggs were transferred to filtered natural seawater (FSW) or FSW containing 0.5 mM ZnSO_4_. Embryos were cultured by standard methods with FSW at 15°C. Cilia were isolated from embryos by treatment with 2X ASW to double the salt concentration [Auclair and Siegel, 1966]. Embryos were first removed by a swing-type of centrifuge (TOMY LC122, TOMY, Tokyo, Japan) at 1,800 rpm for 1 min, and the supernatant was further centrifuged at 2,500 rpm for 10 min to remove embryonic debris. Cilia were collected by centrifugation of the resulting supernatant at 10,000 *g* for 10 min. Successive extraction of ciliary protein was carried out according to Inaba et al. (1988). For isolation of apical tuft from normal embryos, 26-h embryos were treated with 4 μM DD1 [Semenova et al., 2008] for 3 h on ice. After collection of detached cilia, which contained both lateral motile cilia and a part of apical tuft, by centrifugation, the embryos were treated with 2X ASW to collect apical tuft.

### Proteomics

Ciliary proteins (∼25 μg) from normal and Zn-treated embryos at 24- and 36-h post fertilization, respectively, were separated by 2DE. Protein spots stained by SYPRO RUBY were cut out, digested by trypsin, and subjected to peptide mass finger printing with MALDI-TOF/MS (Bruker Daltonics, Billerica, MA) as described [Nakachi et al., 2011].

For the liquid chromatography-tandem mass spectrometry (LC-MS/MS), ciliary proteins were separated by SDS-PAGE with 9% polyacrylamide in the separating gel. Protein loaded on a lane of SDS-gel was adjusted to 25 μg for each sample. Proteins were visualized by Coomassie Brilliant Blue R-250 and excised to 16 pieces per lane. Each piece of the gel was digested with trypsin for the LC/MS/MS analysis as described [Yamada et al., 2009]. The digested peptides were analyzed using a liquid chromatography system (UltiMate® 3000, DIONEX) and MS/MS (LTQ-XL, Thermo Scientific, Rockford, IL).

Raw spectra data were processed using SEQUEST software to extract peak lists. The obtained peak lists were analyzed using the MASCOT program against the *S. purpurutus* protein database extracted from SpBase (http://www.spbase.org/SpBase/). We loaded the same amount of proteins (25 μg) for each of normal or Zn-treated embryos but did not normalize them by taking a certain ciliary protein as a standard. The quantities of the proteins identified by LC-MS/MS were comparable to the peptide counts, which were compared between cilia from normal and Zn-treated embryos. Proteins with a peptide count over two in either normal or Zn-treated embryos were treated as identified in this study. The quantities (peptide count) of the proteins identified by LC-MS/MS were compared between cilia from normal and Zn-treated embryos.

### Isolation of cDNA for GSTT in *H. pulcherrimus*

A part (660 bp) of the protein-coding region of GSTT cDNA was amplified from an *H. pulcherrimus* mesenchyme blastula cDNA library by PCR using following primers: GSTT-F1, ATGACAATCCAGCTGTACGTT; GSTT-R1, CTACTTCGCAAGCGAATCTCT. The 5′- and 3′-UTRs were amplified by rapid-amplification of cDNA ends. The sequence of the full-length cDNA was deposited to DDBJ/EMBL/NCBI (Accession number, AB762295).

### Whole-Mount in situ Hybridization

Whole-mount in situ hybridization was performed as described [Minokawa et al., 2004; Yaguchi et al., 2010]. cDNA containing the protein coding region was reverse-transcribed by T7 polymerase (Takara Bio, Shiga, Japan) with nucleoside triphosphate (NTP) containing digoxygenin-labeled uridine triphosphate (UTP). Embryos were fixed with 4% paraformaldehyde in FSW 4°C overnight. After extensive washing with a MOPS [3-(N-morpholino)propanesulfonic acid] (MOPS) buffer (0.1 M MOPS, pH 7.0, 0.5 M NaCl, 0.1% Tween-20), embryos were further washed with a hybridization buffer [70% formamide, 0.1 M MOPS, pH 7.0, 0.5 M NaCl, 1.0 mg/mL bovine serum albumin (BSA), 0.1% Tween-20]. Prehybridization and hybridization with probes was carried out at 50°C for 3 h and 1 week, respectively.

### Antibodies

The protein-coding region of GSTT was subcloned into pET32a vector and transfected into *Escherichia coli* AD494. Protein expression was induced by 1.0 mM iso-propoly-β-D-thiogalactoside. The purification of fusion proteins and the preparation of polyclonal antibodies in mouse were carried out as described [Padma et al., 2003; Mizuno et al., 2009].

### Analysis of Ciliary Motility and Embryonic Behavior

The ciliary movements were observed and analyzed as described [Shiba et al., 2002; Yaguchi et al., 2010]. The embryos were observed under a phase contrast microscope (BX51; Olympus, Tokyo) equipped with a high-speed camera (200 frames per sec, HAS-220; Ditect, Tokyo, Japan). The embryos at 24–30-h postfertilization were immobilized between a slide glass and a coverslip separated by 58-μm-thick mending tape (3M Scotch). Cilia of the apical tuft and those in the lateral region of embryos were observed and analyzed. Individual images of ciliary movements were analyzed with Bohboh software (Bohboh Soft, Tokyo, Japan). We defined the angles of apical tuft cilia as that between the animal-vegetal axis of the embryo and the straight line from the base to the tip of the cilium. We estimated the shear angle as the angle of the tangent to the ciliary shaft measured with respect to the direction of the axis of the ciliary base.

We recorded the swimming behavior of sea urchin embryos with a stereomicroscope (MZ12.5; Leica, Tokyo) equipped with a digital camera (HDR-CX700; Sony, Tokyo). The embryos at 24–30-h postfertilization were kept in seawater in a chamber by a glass slide (1% BSA-coated) and coverslip sandwiched with a 1-mm-thick silicon spacer. The swimming behaviors of embryos in the chamber were observed at room temperature. For the analysis of geotactic behavior and escaping behavior, a chamber or a micro-maze similarly made of silicon spacers as described above was vertically placed. Embryos were inserted by a pipette at a small entrance opened at the base of the spacer. Swimming velocities and rotational direction were analyzed with Bohboh software. The images of embryonic distribution in a chamber were taken from the movie and processed by Photoshop and Bohboh software.

### Electron Microscopy

Embryos were fixed in a solution (0.45 M sucrose, 2.5% glutaraldehyde, 0.1 M sodium cacodylate; pH 7.4) at 4°C for 2 h. After three washes with 0.45 M sucrose buffered with 0.1 M sodium cacodylate (pH 7.4), the embryos were postfixed with 1% OsO_4_ buffered with 0.1 M sodium cacodylate (pH 7.4) on ice for 2 h. They were then washed with 0.1 M sodium cacodylate (pH 7.4) at 4°C for 10 min, dehydrated through an ethanol series, and embedded in Quetol 812 (Nisshin EM, Tokyo, Japan). The resin was solidified sequentially at 37°C overnight, 45°C for 12 h, and 60°C for 48 h and thin-sectioned with an average thickness of 70 nm. Sections were stained with uranyl acetate and observed under a transmission electron microscope (JEM 1200EX; JEOL, Tokyo, Japan).

### Computational Analysis

Protein identification from mass spectra was done using Mascot (Matrix Science, Boston, MA). The translation of DNA sequences into amino acid sequences, the calculation of molecular mass, the design of PCR primers, and the estimation of isoelectric points were done with GENETYX software. The BLASTP program was used to search for homologous proteins. Multiple sequences alignment and drawing of phylogenic trees were carried out by the Neighbor-Joining method using MEGA5 [Tamura et al., 2011].

The accession numbers for GSTs (shown in Fig. 4) from human, mouse and rat are as follows: human alpha1 (NP665683); human mu1 (AAH24005); human pi1 (AAH10915); human omega1 (AAH00127); human kappa1 (AAH50715); human theta1 (NP000844); human theta2 (NP000845); human zeta1 (NP665877); mouse alpha1 (NP032207); mouse mu1 (NP034488); mouse pi1 (NP038569); mouse omega1 (NP034492); mouse kappa1 (NP083831); mouse theta1 (NP032211); mouse theta2 (NP034491); mouse theta3 (NP598755); mouse theta4 (NP083748); rat alpha1 (NP058709); rat mu1 (NP058710); rat pi1 (AAH58440); rat omega1 (NP001007603); rat kappa1 (NP852036); rat theta1 (NP445745); rat theta2 (NP036928); rat theta3 (NP00113115); human sigma (EAX06052); mouse sigma (NP_062328); rat sigma (NP_113832). Protein sequences used for *S. purpuratus* GSTs were obtained from SpBase (http://www.spbase.org/SpBase/) and are indicated as SPU numbers.
