# Supplementary figures and images for: Glutathione transferase theta in apical ciliary tuft regulates mechanical reception and swimming behavior of Sea Urchin Embryos
Source: Cytoskeleton (Hoboken). 2013 Aug 19;70(8):453–70. doi: 10.1002/cm.21127 (PMC3812683; doi:10.1002/cm.21127)

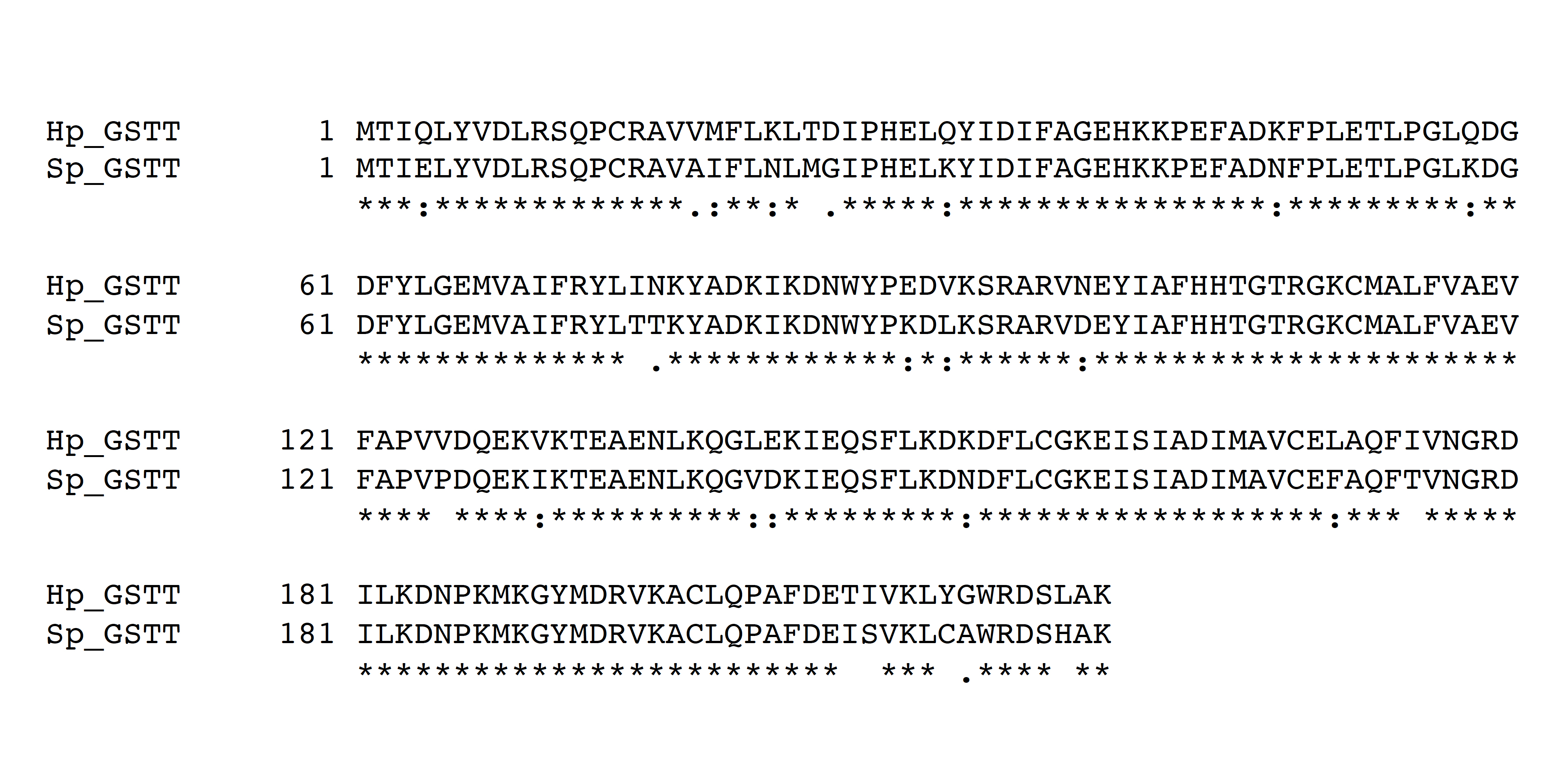

Supplement: Supplementary file 1 [file cm0070-0453-sd1.jpg]
