# Supplementary material for: Glutathione transferase theta in apical ciliary tuft regulates mechanical reception and swimming behavior of Sea Urchin Embryos
Source: Cytoskeleton (Hoboken). 2013 Aug 19;70(8):453–70. doi: 10.1002/cm.21127 (PMC3812683; doi:10.1002/cm.21127)
Supplement: Supplementary file 2 [file cm0070-0453-sd2.pdf]

Table S1. Proteins specific or more abundant in cilia from normal embryos.

Gene ID	Molecular weight	pI	Description	peptide counts in normal embryo	peptide counts in Zn-treated embryo	N/Z
SPU_011586	62619.5	7.98	aminotransferase class V-1	24	-	-
SPU_022860	136675.3	6.85	ankyrin 1-3	8	-	-
SPU_001025	69496.8	9.56	hypothetical protein-321	8	-	-
SPU_013999	61453.2	8.55	ankyrin 1-2	7	-	-
SPU_026386	40267.2	6.71	alcohol dehydrogenase class 3	6	-	-
NP_999633.1	75453.5	6.36	77 kDa echinoderm microtubule-associated protein	5	-	-
SPU_014343	96739.4	5.58	Iron-responsive element binding protein 1	5	-	-
SPU_000974	53865.5	6.54	coronin	5	-	-
SPU_028435	34187	10.17	hypothetical protein-2955	5	-	-
SPU_006911	75632.7	6.36	77kDa-microtubule-associated protein	5	-	-
SPU_022935	39144.1	9.28	unknown Ser/The protein kinase	5	-	-
SPU_027911	16443.7	5.46	FH1/FH2 domain-containing protein 3-like	5	-	-
SPU_004248	48274.1	4.45	clusterin associated protein 1	4	-	-
SPU_028633	28321.2	5.65	Glutathione transferase omega 1 (GSTO 1-1)	4	-	-
SPU_026711	27642.4	5.61	proteasome subunit, alpha type 3	4	-	-
XP_001188734.1	6557.6	9.36	similar to ENSANGP00000021736, partial	4	-	-
SPU_026035	39968.6	5.39	leucine rich repeat containing 34-1	4	-	-
SPU_010970	22267.1	9.54	hypothetical protein isoform 1	15	1	15
SPU_010981	77300.6	5.37	arachidonate 5-lipoxygenase-4	14	1	14
SPU_006747	59730.7	9.58	family with sequence similarity 154, member B	10	1	10
SPU_002011	96337.4	5.22	doublecortin domain containing 2	10	1	10
SPU_025011	103635.9	4.86	hypothetical protein-1134	8	1	8
SPU_021463	59717.3	6.53	IFT 144 (Chlamydomonas reinhardtii)	8	1	8
SPU_010980	47817.1	5.41	arachidonate 5-lipoxygenase-6	24	4	6
SPU_018582	29056.2	9.03	cytoplasmic dynein 2 light intermediate chain 1	12	2	6
SPU_000074	36013.9	8.75	41 kDa centrosomal protein	6	1	6
XP_001185605.1	19993.4	8.93	hypothetical protein, partial	6	1	6
SPU_005282	66085.2	8.05	human chromosome 14 open reading frame 166B	15	3	5
SPU_025893	67824.3	9.75	similar to OTTHUMP00000016025	5	1	5
SPU_019742	37610.6	8.08	calpain 9; calcium-dependent cysteine proteinase	5	1	5

Proteins with peptide counts from mass spectrometry more than 4 and N/Z more than 5 are listed.
